# Editorial: One Step at a Time: Advances in Osteoarthritis

**DOI:** 10.3389/fvets.2021.727477

**Published:** 2021-07-16

**Authors:** Ali Mobasheri, Troy N. Trumble, Christopher R. Byron

**Affiliations:** ^1^Research Unit of Medical Imaging, Physics and Technology, Faculty of Medicine, University of Oulu, Oulu, Finland; ^2^Department of Regenerative Medicine, State Research Institute Centre for Innovative Medicine, Vilnius, Lithuania; ^3^Departments of Orthopedics, Rheumatology and Clinical Immunology, University Medical Center Utrecht, Utrecht, Netherlands; ^4^Department of Joint Surgery, First Affiliated Hospital of Sun Yat-sen University, Guangzhou, China; ^5^World Health Organization Collaborating Center for Public Health Aspects of Musculoskeletal Health and Aging, Université de Liège, Liège, Belgium; ^6^Veterinary Population Medicine, University of Minnesota Twin Cities, St. Paul, MN, United States; ^7^Department of Large Animal Clinical Sciences, Virginia-Maryland College of Veterinary Medicine, Virginia Tech, Blacksburg, VA, United States

**Keywords:** osteoarthritis, humans, companion animals, articular cartilage, synovium, inflammation, subchondral bone

Osteoarthritis (OA) is the most common form of arthritis^1^ and the fastest growing cause of disability worldwide (1, 2). OA is a degenerative and low-grade inflammatory disease that affects humans, companion and captive animals (3). For many years OA was considered a non-inflammatory disease of articular cartilage in elderly individuals and studied in the context of aging (4–6). However, despite the fact that aging is the primary risk factor for OA (7), there is emerging evidence to suggest that OA is a low-grade inflammatory disease that affects the whole joint (8), impacts younger people (9) and involves innate immunity (10, 11). Loss of articular cartilage has long been considered a hallmark of OA (12). However, all the connective tissues that make up a synovial joint have been shown to be altered during the development and progression of OA (6). Cartilage, synovium, subchondral bone, and the crosstalk between these tissues are central components of OA development (13). However, emerging evidence suggests that other tissues such as the meniscus, cruciate ligament, and infrapatellar fat pad, also known as Hoffa's fat pad, are involved in knee OA (14–19).

Low-grade inflammation (20, 21) and metabolic alterations (22–24) occur as individuals age and joint tissue accumulate damage and undergo senescence over time (25, 26), but this does not mean that OA is simply a “wear and tear” disease. Indeed, describing OA as “wear and tear” has hampered research progress and drug development (27, 28). The interplay among diverse cells and their microenvironment which becomes increasingly inflammatory as the disease progresses, as well as the diverse biomechanical and molecular factors of different joints (29, 30) are all active co-conspirators in making this disease so complex and difficult to treat. Although age, obesity, and a previous history of joint trauma are important risk factors for the development of OA (31–33), there is not a single common risk factor defining the origin of the disease, making it even more difficult to define, diagnose, and manage (34). In fact, even when the origin is similar, progression is usually not predictable because of individual variation in metabolic status, fitness level, lifestyle choices, and pain tolerance. To complicate matters even further, there are underlying disease endotypes (35) and emerging phenotypes (36), which add to the complexity of OA. However, the advent of OA endotypes and phenotypes have created new opportunities for stratification and subtyping, allowing us to develop targeted treatments, for example, new therapies for the inflammatory phenotype of the disease (37). There is currently no cure or treatment for OA making effective management extremely challenging (38, 39). This is especially important for an increasingly younger group of individuals who have to live with OA and joint pain for more years and suffer disability and a limited range of motion for most of their lifetime (40). To date, most treatments focus on palliative relief of symptoms. Non-steroidal anti-inflammatory drugs (NSAIDs) are often effective for relief of signs of musculoskeletal pain, however systemic adverse side-effects remain a major concern (41, 42). Furthermore, NSAIDs are not suitable for use in patients with cardiovascular co-morbidities (43, 44) and especially those with chronic asthma (45). Targeted treatment via intra-articular administration of corticosteroids is also palliative but can have undesired consequences for joint tissues (46), although experimental conditions can alter conclusions about the true importance of these effects (47, 48). Results of studies regarding effects of orthobiologic treatments for OA are promising but long-term outcomes remain unknown and require larger and more comprehensive clinical trials with appropriate endpoints.

Therefore, it is paramount that the OA research community continues to strive toward a better mechanistic understanding of the disease. This is the only strategy that can reveal novel therapeutic targets and facilitate the development of earlier diagnostics so that the disease can be identified and managed as early as possible to minimize structural and symptomatic progression. The ultimate goal should be to halt and reverse the progression of OA, but in the absence of such treatments any preventive strategy that can provide effective management such that the joint is not only protected, but that it also has the capability to heal to the best of its ability would support the concept of joint health maintenance (49).

OA is not specific to just one species as veterinary and human patients alike are afflicted by this naturally-occurring disease. This provides opportunity for both; for instance, there is evidence that research in dogs can reliably predict treatment efficacy in humans and vice versa. Across many classes of anti-inflammatory and analgesic compounds in which there have been studies in companion animal chronic pain conditions and the same conditions in humans, analogous results have been seen (3). This has helped advance the knowledge base about the disease process because important comparisons can be made between species. There are numerous animal models of OA and although many can advance our knowledge of disease mechanisms and the pathophysiology of pain (50), none of the small animal models are as relevant to humans as the spontaneously occurring canine and equine models of OA. Indeed, because of the complexity of OA, such spontaneous models of naturally-occurring disease may offer the best way forward for elucidation of pathophysiologic mechanisms and discovery of treatments (51). Nevertheless, the multitude of spontaneous and induced animal models of OA have allowed both physicians and veterinarians to understand the underlying mechanisms and develop strategies that can help decrease the morbidity of the disease for their patients and advance clinical trials of new OA treatments (52).

Much of what we know about OA comes from large-scale epidemiological studies (53–56), especially studies that were conducted in large cohorts such as the Framingham Study (57, 58). Many investigators have designed and conducted clinical trials to examine the effects of physical activity and inactivity on synovial joint health and OA symptoms (59). In humans obesity and lack of physical activity are major contributors to the development of OA (57). There is no convincing published evidence in humans that exercises such as running contributes to OA (60, 61). Recent clinical research suggests that weight loss, physical activity, and increasing muscle mass and strength are the only effective strategies for reducing pain and enhancing mobility in subjects with OA (62). The only way to slow down the pain and progression of OA appears to be physical exercise, avoiding obesity and maintaining a healthy weight (63–69). A recent systematic review of the published literature on studies of the senolytic effects of exercise and physical activity on senescent cells under various states in both human and animal models suggests that exercise has senolytic properties (70). There is also emerging evidence that exercise can support the immune system and generate immune cells through its actions on bone (71). Therefore, these recent observations may explain the beneficial impacts that patients with OA see when they exercise. Reduced levels of physical activity can accelerate the development of OA (72). Therefore, the physical activity that has been recommended for human patients with OA can also be recommended for companion animals, who need it as much as we do.

It is important to pause and reflect on where we are going, which areas we should focus on in the future and which topics might greater effort in this field. The study of extracellular vesicles (ECVs) in OA has become a whole new area (73–75). Epigenetics, epigenomics and the study of microRNAs are also rapidly expanding and thriving areas of research in the OA field (76, 77). Advances in analytical platforms such as omics technologies for deep phenotyping (28, 78), biomarkers (79, 80), structural and functional imaging (81, 82) and artificial intelligence (83, 84) are also having an impact on our understanding of OA. Continuing technological advances that impact clinical and laboratory diagnostics are making it possible to investigate this disease in ways that were impossible to do just a few decades ago. This includes, but is not limited to, learning more about the basic biology of joint tissues and how they can be stimulated to repair or be replaced, how the disease can be diagnosed earlier using biological or imaging biomarkers, as well as identifying and testing various therapeutic targets.

The authors strongly believe that future advances in OA research require multidisciplinary and interdisciplinary collaboration and a genuine “One Health,” “One Medicine” approach to OA, with closer interaction between veterinarians, human clinicians and bioscience researchers (85).

This Research Topic comprises 4 review articles and 6 original research publications from a number of OA researchers. Taken together, these articles are geared toward the advancement of our understanding, diagnosis, and treatment of OA by researching multiple intra-articular tissues including synovial membrane, articular cartilage, subchondral bone, and fat. Multiple technologies are used in the original research articles to determine changes that occur with disease and/or treatment using novel *in vitro* methods, animals with naturally-occurring OA or post-traumatic OA models that have translational relevance to human OA.

## Review Articles

Regenerative medicine and cell-based therapies are promising areas of research and development in many disease areas, including OA. These therapies have the potential to provide symptomatic relief while also potentiating repair. In this Research Topic, 2 reviews are presented on this area as it relates to veterinary medicine. Bogers reviews the known mechanism of action of mesenchymal stem cells, and the blood derived products autologous conditioned serum (ACS) and platelet-rich plasma (PRP) for canine and equine OA patients. The review explores current preclinical and clinical efficacy in joint disease in the context of the processing type and study design, as well as the regulatory aspects that need to be considered when administering cell-based therapies. Garbin and Morris focus their review on ACS and autologous protein solution hemoderivatives that produce high concentrations of Interleukin-1 receptor antagonist (IL-1Ra) and other cytokines and growth factors that can modulate OA effects and progression. The review compares and contrasts them with each other focusing on the clinical and modulatory effects as well as the limitations of use in equine OA patients.

Knowledge of the interplay of other articular tissues besides articular cartilage and synovium on the onset and progression of OA continues to expand with the rapid and frequent advancement of imaging technology such as computed tomography (CT) and magnetic resonance imaging (MRI). For instance, changes to intra-articular fat pads have increasingly been highlighted in MRIs of OA patients. Labusca and Zugun-Eloae discuss the role of intra-articular adipose tissue, such as Hoffa's fat pad in the knee, in the homeostasis of synovial joints and the pathogenesis of joint pathologies such as OA. They state that even though the structure is similar to subcutaneous adipose tissue, the molecular regulation of intra-articular adipose tissue is different, and they propose that articular fat pads are an active component of the joint with multiple functions and important roles in the maintenance of tissue homeostasis. Subchondral bone changes have also been highlighted in multiple species with OA by advanced imaging. Stewart and Kawcak review the current understanding of the role of subchondral bone in OA. They explore the importance of studying the osteochondral unit and the relationship between subchondral bone and OA across veterinary species, but their focus is on equine, arguably one of the most suitable biomechanical models for the study of OA. They detail recent progress in advanced imaging for the diagnosis of early disease and provide thoughts on treatment and prevention.

## Original Research Articles

### *In vitro* Studies

Byron and Trahan evaluated a novel *in vitro* co-culture system for investigating new and existing OA treatments. This co-culture system was comprised of equine osteochondral and synovial explants and they compared it to traditional equine articular cartilage explants. Both systems were stimulated with Interleukin-1beta (IL-1beta) and their results suggest similar outcomes with some important differences between culture systems in their response to inflammatory stimuli. In particular, the co-culture system was able to significantly dampen the increase in the degradative enzyme matrix metalloproteinase-13 compared to the cartilage explant. Since the synovium is a key contributor to a joint environment, these findings suggest that the co-culture system may be more relevant to *in vivo* physiology than traditional *in vitro* articular cartilage explants alone.

Concentrations of PRP are subject to variability from the patient itself as well as the processing method. Therefore, Gilbertie et al. set out to examine the effect of a pooled allogenic platelet-rich plasma lysate (PRP-L) as an alternative therapy that might decrease variability since preparations of PRP-L are acellular, contain high concentrations of growth factors and cytokines, and can be stored for immediate use. The effects of PRP-L were evaluated on equine synoviocytes and chondrocytes challenged with pro-inflammatory mediators *in vitro*, in a model that mimics the inflammatory micro-environment in the OA joint. The results showed a protective effect of PRP-L mostly through an increase in anti-inflammatory cytokines rather than a reduction of pro-inflammatory mediators, demonstrating a need for further studies on the use of pooled PRP-L for the treatment of OA.

### Models of OA (Equine Naturally-Occurring and/or Post-traumatic)

In synoviocytes and chondrocytes galectins are potent regulators of cell adhesion, growth, and apoptosis, however the role of galectins-1 and-3 are unknown in OA. Therefore, Reesink et al. set out to investigate these galectins in naturally-occurring OA as well as in a post-traumatic osteochondral injury model in horses. They demonstrated that both are present in healthy synovial fluid, and that concentrations increase after osteochondral fragmentation. Furthermore, galectin-3 staining was found around healthy superficial chondrocytes whereas galectin-1 staining was limited to chondrons and injured cartilage. Their work demonstrates a possible role in OA, but further research is needed.

Macrophage phenotypes in synovium from healthy and OA joints are poorly characterized. Menarim et al. set out to compare the patterns of activation of M1-like and M2-like macrophage phenotypes in healthy and naturally-occurring OA equine joints with comparison to histology and cytokine/chemokine profiles in synovial fluid. Their results demonstrated that all macrophage markers were proportionate to the degree of synovial inflammation, with minimal difference between OA and normal joints. They emphasized that equine synovial macrophages are in a hybrid state of activation that display a regulatory response that targets the resolution of inflammation, but point out that further study is needed to see if this homeostatic response can be maximized.

### Response to Therapeutics

While intra-articular administration of corticosteroids, hyaluronan, or a combination of both have been commonly used in equines and humans, they are not used commonly in canines. Alves et al. sought to determine if a single dose of Triamcinolone Acetonide, hyaluronan, or a combination of both would be safe and effective at decreasing pain in working police dogs with naturally-occurring hip OA. The results demonstrated that all 3 treatment arms were safe and effective, but the combination was the only one that demonstrated significant improvement on multiple visual pain scales. Future studies are needed with larger sample sizes, different doses, and administration frequency.

Cannabidiol (CBD) is a non-competitive antagonist of cannabinoid receptors with potential immunomodulatory, anti-hyperalgesic, anti-nociceptive, and anti-inflammatory actions. Gamble et al. carried out a clinical study to determine the basic pharmacokinetics, safety, and analgesic efficacy of a CBD-based oil in dogs with radiographic OA. Their study suggests that CBD is bioavailable, with no observed side effects. They noted that 2 mg/kg of CBD twice daily was well-tolerated and appeared to increase physical activity in dogs with OA. The long-term effects of CBD still need to be determined.

We have enjoyed editing this Research Topic for Frontiers in Veterinary Science and sincerely hope that readers will enjoy reading these significant contributions that remind us of the crucial importance of interdisciplinary collaboration between those working on OA in human medicine and their counterparts in veterinary medicine. Future progress will be significantly enhanced if these communities communicated and collaborated more effectively.

## Author Contributions

All authors listed have made a substantial, direct and intellectual contribution to the work, and approved it for publication.

## Conflict of Interest

The authors declare that the research was conducted in the absence of any commercial or financial relationships that could be construed as a potential conflict of interest.

## Footnotes

^1^https://www.cdc.gov/arthritis/basics/osteoarthritis.htm.
